# Efficacy of dacomitinib in patients with EGFR‐mutated NSCLC and brain metastases

**DOI:** 10.1111/1759-7714.14222

**Published:** 2021-11-09

**Authors:** Jinyao Zhang, Yan Wang, Ziling Liu, Lin Wang, Yu Yao, Yutao Liu, Xue Zhi Hao, Jianyang Wang, Puyuan Xing, Junling Li

**Affiliations:** ^1^ Department of Medical Oncology, National Cancer Center/National Clinical Research Center for Cancer/Cancer Hospital Chinese Academy of Medical Sciences and Peking Union Medical College Beijing China; ^2^ Department of Oncology Center The First Hospital of Jilin University Changchun China; ^3^ Department of Medical Oncology The First Affiliated Hospital of Xi'an Jiaotong University Xi'an China; ^4^ Department of Radiotherapy, National Cancer Center/National Clinical Research Center for Cancer/Cancer Hospital Chinese Academy of Medical Sciences and Peking Union Medical College Beijing China

**Keywords:** brain metastasis, dacomitinib, EGFR, EGFR TKI, NSCLC

## Abstract

**Background:**

Dacomitinib is a second‐generation epidermal growth factor receptor (EGFR) tyrosine kinase inhibitor (TKI) which is superior to first‐generation EGFR TKI in ARCHER 1050. However, the activity of dacomitinib in the central nervous system (CNS) is not known as ARCHER 1050 did not include patients with baseline brain metastases. This study aimed to describe dacomitinib's activity in the CNS in a real‐world setting.

**Patients and Methods:**

Thirty‐two patients who were receiving dacomitinib for advanced non‐small‐cell lung cancer (NSCLC) with EGFR mutations and brain metastasis were included in this study. Patients who received prior EGFR TKIs were excluded from this trial. Case report forms were collected to determine treatment outcomes.

**Results:**

Among 32 patients with EGFR‐mutated NSCLC and brain disease, eight were included in the CNS evaluable for response group. The intracranial objective response rate (iORR) was 87.5% (95% confidence interval [CI] 47.3–99.7%) and the intracranial disease control rate (iDCR) was 100% (95% CI 63.1–100%). In 30 evaluable patients with measurable or nonmeasurable brain lesions, the iORR was 66.7% (95% CI 47.2–82.7%) and the iDCR was 100% (95% CI 88.4–100%). Median intracranial duration of response (iDoR) and intracranial progression‐free survival (iPFS) were not reached, with a one‐year iDoR rate of 72.2% (95% CI 48.7–95.7%) and a 1‐year iPFS rate of 71.2% (95% CI 51.0–91.4%), respectively. The majority of patients experienced low‐grade (G1/2) toxicities, which are reversible.

**Conclusion:**

This study suggests that dacomitinib demonstrated CNS efficacy in patients with EGFR TKI‐naïve EGFR‐mutated NSCLC in the real‐world setting. The safety profile was tolerable and manageable.

## Graphical abstract

This study is the largest multicentric trial to demonstrate the efficacy of dacomitinib in NSCLC patients with EGFR mutation and brain metastases. The survival time of NSCLC patients with EGFR mutation and brain metastases could be prolonged with dacomitinib treatment.

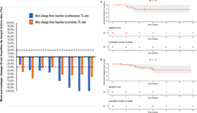

## INTRODUCTION

Brain metastases (BMs) are a common site for non‐small‐cell lung cancer (NSCLC) and create a challenge for therapy.^1^ The incidence of BMs has been observed around 25% at first diagnosis in patients with EGFR‐mutated NSCLC.^2^ The standard of care for BMs includes surgical resection, stereotactic radiosurgery (SRS), and whole‐brain radiation therapy (WBRT). However, patients with BMs had a poor prognosis despite treatment.^3^ In recent decades, the treatment landscape for patients with NSCLC has changed with the approval of tyrosine kinase inhibitors (TKIs), leading to long‐term survival, especially in patients with EGFR mutation.^4^ Nevertheless, due to the limited ability of EGFR TKIs to penetrate the blood brain–barrier (BBB), the cumulative risk of developing brain metastases increases to more than 45% at 3 years after initial diagnosis.^2^ As the incidence of BMs increases, there is an increasing need to establish an optimal strategy for them.

Dacomitinib is an oral, irreversible pan‐ErBb inhibitor and has been approved for patients with EGFR‐sensitive mutated NSCLC as first‐line treatment. A phase III clinical trial, RCHER 1050, compared dacomitinib to gefitinib in untreated patients with EGFR‐mutated NSCLC and randomly assigned patients to dacomitinib or gefitinib by a ratio of 1:1. Dramatically, a significant difference was observed between the two arms. The progression‐free survival (PFS) benefit was found to be higher in the dacomitinib arm than in the gefitinib arm (14.7 months vs. 9.2 months, hazard ratio [HR] 0.59, 95% confidence interval [CI] 47–74%, *p* < 0.0001).^5^ In addition, median overall survival (OS) was prolonged in the dacomitinib arm compared with the gefitinib arm, thus dacomitinib is the first target drug to improve overall survival (34.1 months vs. 27.0 months, HR 0.748, 95% CI 59.1–94.7%, *p* = 0.0155).^6^ However, the central nervous system (CNS) response to dacomitinib remains unknown due to excluding patients with BMs in ARCHER 1050.

In a series of LUX lung clinical trials, afatinib, another second‐generation EGFR TKI, demonstrated clinical activity and survival benefit in EGFR‐mutated NSCLC patients with BMs compared to chemotherapy or first‐generation EGFR TKI.^7^, ^8^, ^9^ Likewise, it was found that the risk of developing BM in the dacomitinib arm was lower than in the gefitinib arm in ARCHER 1050, with one patient experiencing CNS progression in the dacomitinib and 11 in the gefitinib arm.^5^ A preclinical test also demonstrated that systemic dacomitinib treatment effectively inhibits tumor growth in glioblastoma (GBM) brain xenografts, indicating that dacomitinib is capable of crossing the BBB.^10^ These findings implied that the second‐generation EGFR TKIs afatinib and dacomitinib have intracranial antitumor activity.

Herein, we aimed to investigate the efficacy of dacomitinib in EGFR‐mutated NSCLC, focusing on untreated patients with brain metastases at baseline.

## MATERIALS AND METHODS

### Patients and data collection

This was a multicentric observational real‐world study with retrospective and prospective data collections to describe the CNS response to dacomitinib (15 mg p.o (peros). od (once daily), 30 mg p.o. od or 45 mg p.o. od) in patients with EGFR‐mutated NSCLC. The study included adult patients with relapsed or metastatic NSCLC harboring EGFR mutations at seven centers after 1 August 2019. The inclusion criteria were (1) pathologically or cytologically confirmed NSCLC, (2) EGFR mutations, (3) patients who had never received EGFR TKI after initial diagnosis, and (4) patients with stable CNS disease or with unstable CNS disease who had received CNS radiotherapy (RT) (include WBRT, SRS, gamma knife, etc.). The exclusion criteria were (1) accompanied by other malignant tumors, (2) lack of clinical information, and (3) combined with other anticancer drugs. Brain computed tomography and magnetic resonance imaging (MRI) weres used to diagnose BM before dacomitinib treatment. Genetic testing by next‐generation sequencing (NGS) or the amplification refractory mutation system (ARMS) method was required to determine EGFR mutations. Only patients with at least one measurable brain lesion on the baseline and no prior CNS RT were included in the CNS evaluable for response (cEFR) group. Patients' clinical information was collected through case report forms (CRFs). The data cut‐off date was 7 July 2021. This study was approved by the institutional review board or independent ethics committee and registered in Clinical trial.gov (number NCT04768491).

### Assessments

The interval of response evaluation was required to be every 1–2 months in the treatment period until treatment discontinuation. The evidence and assessment of BMs are only in accordance with magnetic resonance imaging (MRI). Tumor response was evaluated by the Response Evaluation Criteria in Solid Tumors (RECIST) version 1.1 and the modified RECIST version 1.1,^11^ respectively. In the modified RECIST 1.1, up to five intracranial and up to five extracranial target lesions were included; intracranial target lesions of between 5 and 40 mm in diameter were allowed. The primary endpoint for CNS analysis was intracranial objective response rate (iORR) and intracranial disease control rate (iDCR), and the secondary endpoint was time to intracranial response (iTTR), intracranial duration of response (iDoR), intracranial progression‐free survival (iPFS), and median best percentage change from baseline in target lesion (TL) size. Adverse events were recorded based on the National Cancer Institute Common Terminology Criteria for Adverse Events.

### Statistical analyses

Continuous variables were described as mean, median, standard deviation, minimum and maximum, and categorical variables were described as the frequency and percentage. The definition of iORR was the percentage of patients who had a best intracranial response of complete response (CR) or partial response (PR). Intracranial DCR included intracranial CR, PR, and stable disease (SD). iTTR was defined as the time from the first dose of dacomitinib to the time when the intracranial response to dacomitinib was first observed. iDoR was defined as the time from the first documented intracranial response to the time when CNS progression was observed or dacomitinib treatment failure (by death or extracranial progression in the absence of CNS progression). iPFS was defined as the time from the first dose of dacomitinib until CNS progression or dacomitinib treatment failure (by death or extracranial progression in the absence of CNS progression). The ORR and DCR of the CNS were evaluated with a 95% CI according to the exact binomial distribution. iDoR and iPFS were estimated by the Kaplan‐Meier method with 95% CI. All statistical tests were performed by SPSS version 26.0.

## RESULTS

### Patient characteristics

From 1 August 2019 to 7 July 2021, 110 patients with advanced EGFR‐positive NSCLC received dacomitinib as first‐line treatment in seven centers. A total of 32 patients with brain disease at first diagnosis were included. The baseline characteristics of these patients are given in Table 1. Among these patients, the median age was 57.5 years (range 41–76). Most of them (31/32, 96.9%) were adenocarcinoma, 40.6% of cases were determined by the ARMS method for EGFR mutation types, while 59.4% were identified by NGS method. As for EGFR genotypes, five (5/32, 15.6%) patients were EGFR 19DEL, 25 (25/32, 78.1%) were EGFR L858R, and two (2/32, 6.3%) were uncommon mutations (Table 1 and Supporting Information S1). All patients had never received EGFR TKI. However, six patients had received treatment before dacomitinib, of whom four received one to four cycles of chemotherapy before obtaining gene sequencing results and two had prior CNS RT with at least a 3‐week interval. There were one (3.1%), 28 (87.5%), and three (9.4%) patients started on 15 mg once daily (OD) dacomitinib, 30 mg OD dacomitinib, and 45 mg OD dacomitinib, respectively.

**TABLE 1:** Baseline characteristics of patients

Baseline characteristics (*n* = 32)	Overall (%)
Age, year	
Mean (SD)	59.06 (9.40)
Median (range)	57.5 (41–76)
Brain lesion size (mean [SD]), cm	1.08 (0.85)
Gender, *n* (%)	
Male	13 (40.6)
Female	19 (59.4)
Smoking statue, *n* (%)	
Never	22 (68.8)
Current or former	10 (31.2)
ECOG, *n* (%)	
0–1	28 (87.9)
2	4 (12.1)
Histologic type, *n* (%)	
Adenocarcinoma	31 (96.9)
Other (adenosquamous)	1 (3.1)
Recurrence after surgical therapy, *n* (%)	
Yes	6 (18.8)
No	26 (81.2)
Gene sequencing method, *n* (%)	
ARMS	13 (40.6)
NGS	19 (59.4)
Specimen for gene test, *n* (%)	
Tissue	27 (84.4)
Plasma	3 (9.4)
Pleural effusion	2 (6.2)
EGFR mutation, *n* (%)	
19 del	5 (15.6)
L858R	25 (78.1)
Uncommon	2 (6.3)
L861Q	1 (3.15)
G719A	1 (3.15)
Dose, *n* (%)	
15 mg	1 (3.1)
30 mg	28 (87.5)
45 mg	3 (9.4)
CNS RT, *n* (%)	
No prior brain radiation	30 (93.8)
Prior brain radiation	2 (6.2)
Type of brain RT	
Prior gamma knife	1 (3.1)
Prior WBRT	1 (3.1)
Interval between RT and dacomitinib	
≤4 weeks	1 (3.1)
>4 weeks	1 (3.1)
Prior chemotherapy, *n* (%)	
Yes	4 (12.5)
No	28 (87.5)
Patients with measurable brain lesions, *n* (%)	
Yes	10 (31.3)
No	22 (68.7)
CNS symptom, *n* (%)	
Yes	5 (15.6)
No	27 (84.4)
Peritumoral edema, *n* (%)	
Yes	12 (37.5)
No	20 (62.5)
Size of largest brain lesions, *n* (%)	
<1 cm	22 (68.8)
≥1 cm	10 (31.2)
Number of brain lesions, *n* (%)	
1	13 (40.6)
2–5	8 (25)
>5	11 (34.4)

Abbreviations: ARMS, amplification refractory mutation system; CNS, central nervous system; NGS, next‐generation sequence; RT, radiotherapy; SD, standard deviation; WBRT, whole‐brain radiation therapy.

Patients with more than one brain lesion were common in EGFR + NSCLC (19/32, 59.4%). The mean diameter of the largest brain lesion was 1.08 cm. Five patients (15.6%) had baseline CNS symptoms owing to the CNS disease. Twelve cases (37.5%) had peritumoral edema surrounding brain metastases. At data cut‐off (7 July 2021), the median duration of follow‐up time was 9.4 months (range 2.3–23.3 months).

### Clinical outcome

#### Intracranial efficacy

**FIGURE 1:**
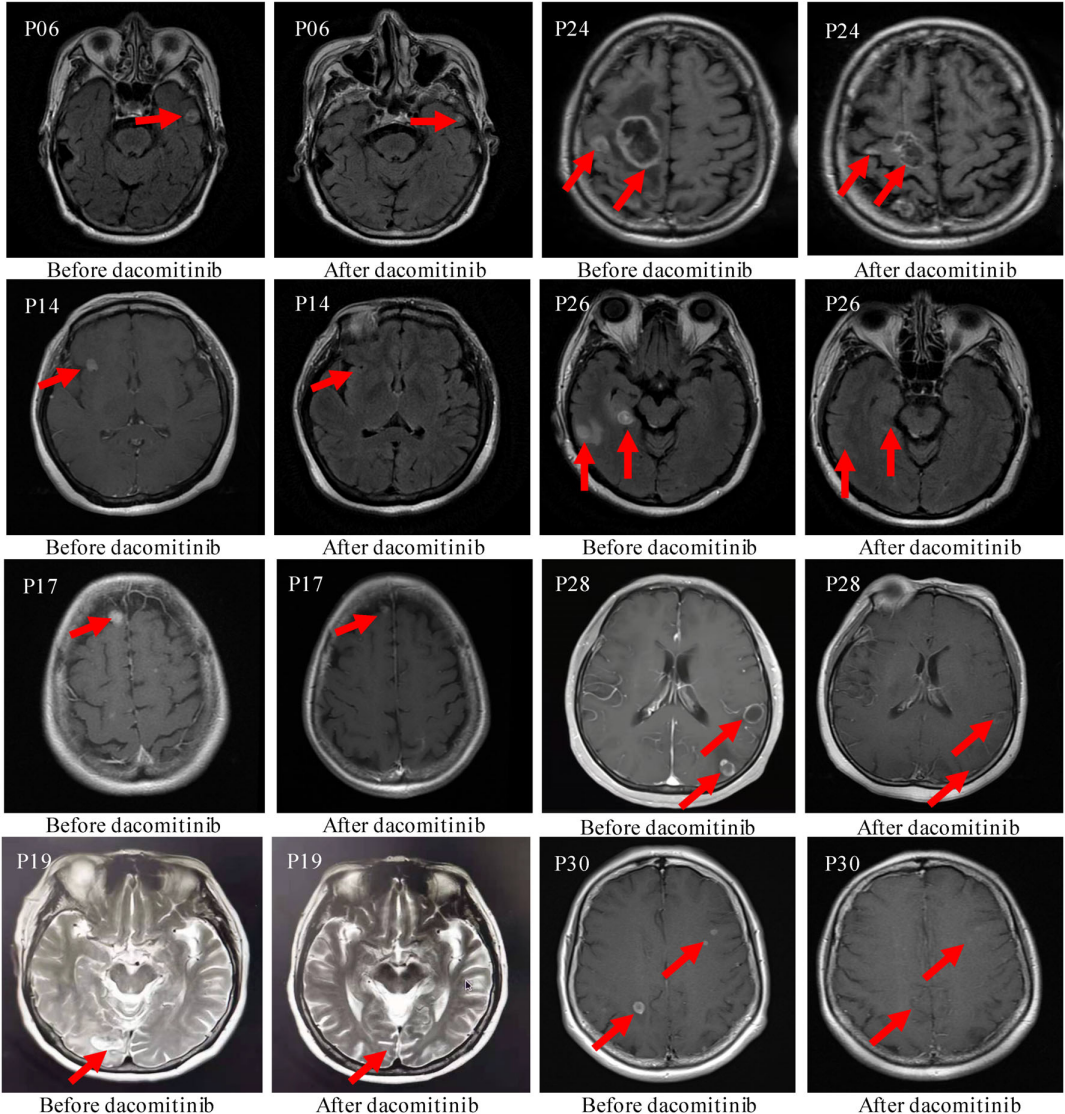
Brain MRI indicating the intracranial response to dacomitinib in patients with measurable lesions

**FIGURE 2:**
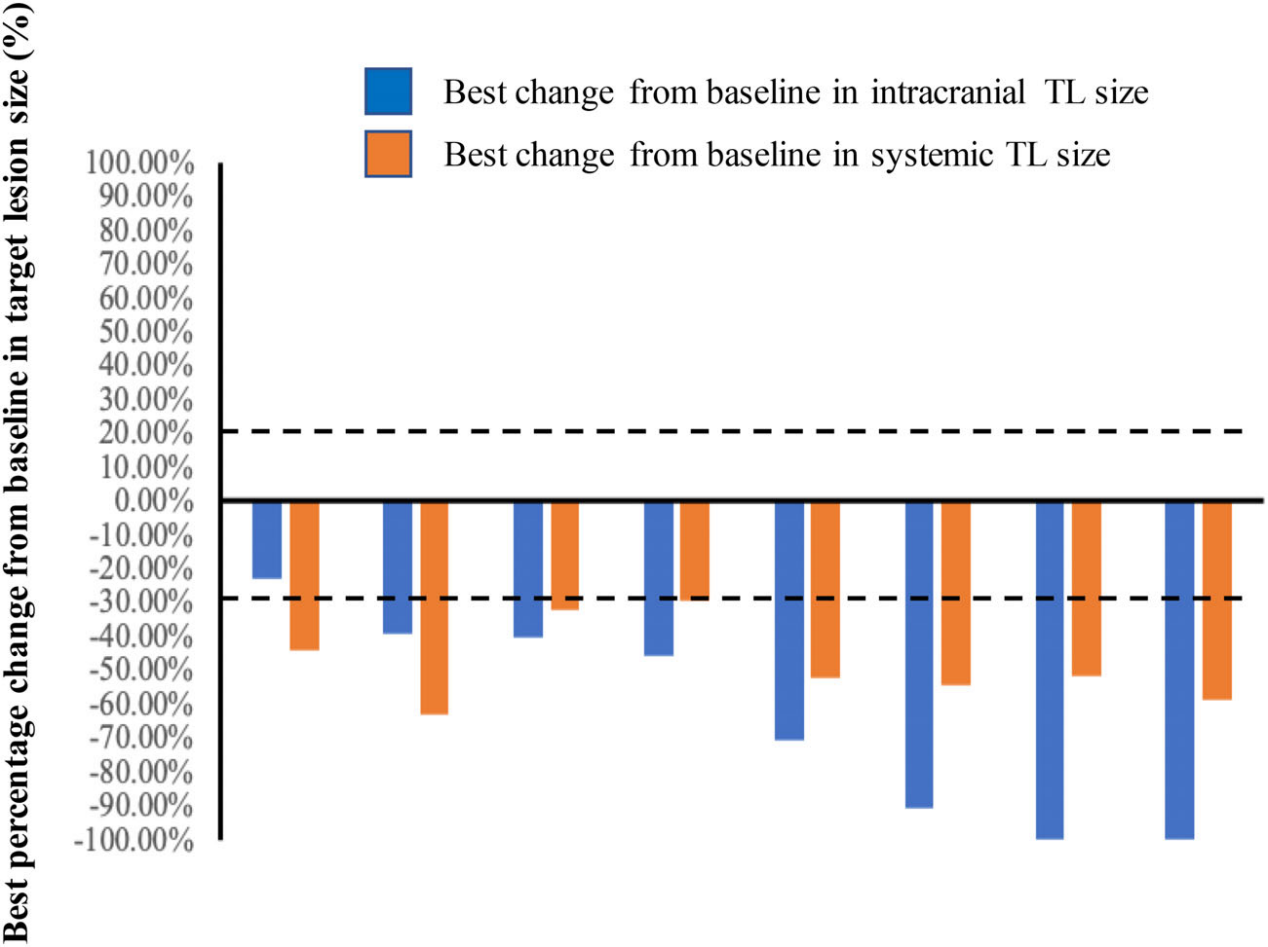
Best percentage change from baseline in target lesion (TL) size

Of 32 patients, eight were included in the cEFR group. In this group, one CR (12.5%), six PR (75%) and one SD (12.5%) were observed, resulting in an iORR of 87.5% (95% CI 47.3–99.7%) and an iDCR of 100% (95% CI 63.1–100%) (Figure 1 and Table 2). The median best percentage change from baseline in TL size was 57.6% (range 22.9–100%) (Figure 2). The median iTTR was 1.03 months (range 0.47–9.4 months) (Table 2). To enhance the accuracy of the CNS tumor response, it was conducted using the modified RECIST 1.1 again and a total of 27 cases were included. The iORR was 85.2% (95% CI 66.3–95.8%), with 12 CR (44.4%) and 11 PR (40.7%), and the iDCR was 100% (95% CI 87.2–100%). This result is consistent with the CNS tumor response for cEFR.

**TABLE 2:** CNS activity of dacomitinib in patients with brain metastases

CNS response to dacomitinib	cEFR (*n* = 8)	All patients (*n* = 30)
Best overall response, *n* (%)		
Complete response	1 (12.5)	14 (46.7)
Partial response	6 (75)	6 (20)
Stable disease or non‐CR/non‐PD	1 (12.5)	10 (33.3)
Progressive disease	0 (0)	0 (0)
Intracranial ORR		
Responder, *n* %	7	20
(95% CI)	87.5 (47.3–99.7)	66.7 (47.2–82.7)
Intracranial DCR		
Patients with brain disease control, *n* %	8	30
(95% CI)	100 (63.1–100)	100 (88.4–100)
Median follow‐up time, month (range)		9.4 (2.3–23.3)
Median iTTR		
Patients included in analysis	7	20
Months (range)	1.03 (0.47–9.4 months)	1.88 (0.77–9.43 months)
Estimated % remaining in response, % (95% CI)		
At 6 months		80.2 (60.0–100)
At 12 months		72.2 (48.7–95.7)
iPFS, % (95% CI)		
Progression free at 6 months		92.4 (82.2–100)
Progression free at 12 months		71.2 (51.0–91.4)

Abbreviations: cEFR, CNS evaluable for response; CI, confidence interval; CNS, central nervous system; CR, complete response; DCR, disease control rate; iTTR, time to intracranial response; ORR, objective response rate; PD, progression disease; iPFS, intercranial progression‐free survival.

**FIGURE 3:**
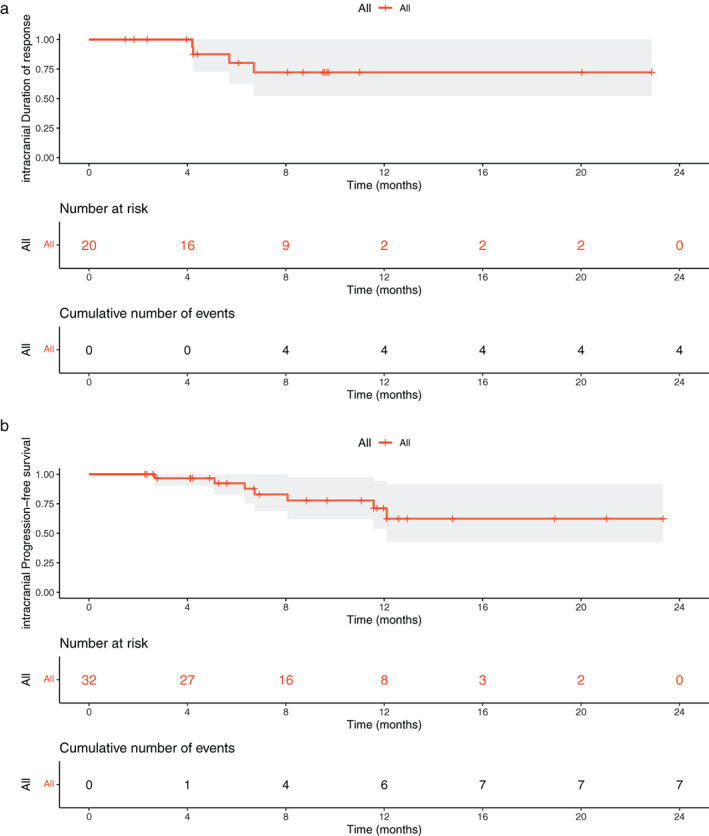
Intracranial duration of response (a) and progression‐free survival (b)

In 30 evaluable patients with measurable or nonmeasurable brain lesions, the iDCR was 100% (95% CI 88.4–100%), with 14 CR (46.7%), six PR (20%), and 10 SD (33.3%). The iORR was 66.7% (20/30, 95% CI 47.2–82.7%) with median iTTR of 1.88 months (range 0.77–9.43 months). Of those patients experiencing a CNS response, median iDoR was not reached after 20% (4/20) of disease progression. The percentage of patients estimated to be remaining in CNS response at 6 and 12 months was 80.2% (95% CI 60.0–100%) and 72.2% (95% CI 48.7–95.7%), respectively (Table 2 and Figure 3a). Of 32 patients included in this study, median iPFS was not reached (95% CI 12.1‐NA), with a 6‐month iPFS rate of 92.4% (95% CI 82.2–100%) and a 1‐year iPFS rate of 71.2% (95% CI 51.0–91.4%) (Table 2 and Figure 3b).

Five patients with neurological symptoms at baseline obviously improved after starting dacomitinib. Peritumoral edema vanished or improved in 91.7% (11/12) of patients.

#### Systemic efficacy

Of 32 patients, 27 with measurable disease were evaluable for systemic response. ORR was 77.8% (95% CI 57.7–91.4%) with PR reported in 21 patients. DCR was 96.3% (95% CI 81.0–99.9%). The median best percentage change from baseline in the sum of overall measurable disease was 44.4% (range 0–100%). The best response of four patients with nonmeasurable lesions was non‐CR/non‐PD (progression disease). The remaining patient was not evaluable for systemic response due to the lack of baseline examination (Table 3). The median PFS in all patients was not reached.

**TABLE 3:** Systemic activity of dacomitinib in patients with brain metastases

Systemic tumor response to dacomitinib	*N* = 32
Complete response, *n* (%)	0 (0)
Partial response, *n* (%)	21 (65.6)
Stable disease, *n* (%)	5 (15.6)
non‐CR/non‐PD, *n* (%)	4 (12.5)
Not evaluable, *n* (%)	1 (3.15)
Progressive disease, *n* (%)	1 (3.15)
ORR, % (95% CI)	77.8 (57.7–91.4)
DCR, % (95% CI)	96.3 (81.0–99.9)

Abbreviations: CI, confidence interval; CR, complete response; DCR, disease control rate; ORR, objective response rate; PD, progression disease.

At data cut‐off, a total of seven patients experienced disease progression: five had extracranial progression only, one had intracranial progression only, and one had both extracranial and intracranial progression (Supporting Information Table S2). The EGFR TKI failure modes were also assessed based on the criteria reported by Yang et al.^12^ Among seven patients with disease progression, three showed dramatic progression, two showed gradual progression, and two showed local progression (Supporting Information Table S2). The overall concordance between brain and systemic response to dacomitinib was 63.3%. Fifteen (15/30, 50%) patients had both a brain and systemic response, and four patients were nonresponders regardless of systematic and brain response (Supporting Information Table S3).

### Safety profile

All patients experienced at least one common adverse event (AE), including rash, stomatitis, diarrhea, dry skin, and paronychia. The majority of AEs were grade 1–2, and death related to severe AE was not reported. However, other AEs occasionally were reported, including asthenia, decreased appetite, and musculoskeletal pain. Seven patients (21.9%) had dose reduction because of intolerable AEs, two of whom started on 45 mg OD. AEs observed during dacomitinib treatment are summarized in Table 4.

**TABLE 4:** Safety profile in dacomitinib‐treated EGFR‐mutated NSCLC with brain metastases

Adverse event	*N* (%)	
	All grades	Grades 3–4
Rash	30 (93.7)	4 (12.5)
Stomatitis	25 (78.1)	1 (3.1)
Diarrhea	27 (84.4)	0 (0)
Paronychia	22 (68.7)	2 (6.3)
Dry skin	24 (75.0)	1 (3.1)
Asthenia	2 (6.3)	0 (0)
Musculoskeletal pain	4 (12.5)	1 (3.1)
Decreased appetite	6 (18.7)	0 (0)
Alopecia	1 (3.1)	0 (0)
Decreased weight	5 (15.6)	0 (0)
Sour regurgitation	2 (6.3)	0 (0)
Nasal mucosal disorder	1 (3.1)	0 (0)
Chilly	2 (6.3)	0 (0)
Increased ALT/AST	1 (3.1)	0 (0)
Nausea	2 (6.3)	0 (0)

### Subsequent therapy

The median duration of dacomitinib treatment was 8.45 months (range 2.3–23.3 months). A total of eight patients discontinued dacomitinib treatment, six owing to disease progression and two due to death or another reason. In addition, two patients received salvage brain radiotherapy while taking dacomitinib. The intervals between the start of dacomitinib treatment and brain radiotherapy were 9.1 and 9.5 months. Of seven patients with disease progression in the dacomitinib treatment period, one patient received bevacizumab treatment and continued to take dacomitinib. The subsequent anticancer treatment in the remaining patients was osimertinib (two cases), chemotherapy combined with bevacizumab (two cases), and dose intensification of dacomitinib (one case) (Supporting Information Table S4).

## DISCUSSION

In this multicentric observational study, dacomitinib demonstrated clinically durable activity and a manageable safety profile in patients with EGFR‐mutated NSCLC and BMs. The results show that dacomitinib has rapid intracranial efficacy, with an iORR of 87.5% and iDCR of 100% in patients with measurable brain lesions. In addition, the safety profile was consistent with that reported in ARCHER 1050, mostly rash, diarrhea, stomatitis, and paronychia, with a low rate of grade 3–4 AEs.

There is limited evidence regarding the efficacy of dacomitinib in brain disease owing to the exclusion of patients with BMs in ARCHER 1050. Currently, the only retrospective study with a small sample size (14 patients) first confirmed the CNS response to dacomitinib, with an encouraging iORR of 85.7% and iDCR of 100% in patients with EGFR‐mutated NSCLC.^13^ However, the data should be used with careful interpretation given the small sample size. Hence, to the best of our knowledge, our research is the largest and first multicentric trial to demonstrate the CNS efficacy of dacomitinib in patients with EGFR mutation. The results of this study support the previous study and may serve as a foundation for further clinical trials. In addition, the role of dacomitinib in patients with BMs from NSCLC is being further investigated in a clinical trial (NCT04339829) carry out by Lu Shun et al.

In our study, iORR and iDCR in the cEFR set were consistent with the previous study reported by Wu Lin et al.^13^ However, in terms of overall population, iORR (66.7%) appears inferior to the previous study. Low iORR in this study was primarily attributed to the short follow‐up time as several patients did not achieve the best response. Of note, although a high percentage of patients had multiple nonmeasurable brain lesions in our study, 14 CR (46.7) was reported. Numerous retrospective studies have described the size and number of BMs in patients with EGFR‐mutated NSCLC. It was found that BMs in NSCLC patients with EGFR mutation frequently manifest as multiple small brain lesions (less than 1 cm).^14^, ^15^ Consequently, our data is convincing and meaningful based on the above findings. Furthermore, given the high proportion of multiple small BMs in EGFR‐mutated NSCLC patients, which leads to intracranial disease assessment difficulties, modified RESCIST 1.1^11^ was applied to evaluate the CNS response to dacomitinib. The results did not differ between the two methods (ORR 87.5% vs. 85.2%), further indicating the reliability of CNS response to dacomitinib. Besides an impressive response rate in our study, the median times to intracranial response in the cEFR set and overall population were 1.03 and 1.88 months, respectively. The majority of patients had responded by the time of the first assessment (4 weeks). Moreover, all patients (5/32) with neurologic symptoms at baseline experienced improvement while taking dacomitinib. The peritumoral edema in most patients had also vanished or improved after taking dacomitinib. These findings indicate that dacomitinib gave EGFR‐mutated NSCLC patients with brain metastases additional treatment options and postponed brain radiotherapy.

In addition to the current study with dacomitinib, several clinical trials or retrospective studies have shown that other EGFR TKIs, including afatinib and dacomitinib, can improve clinical outcomes in EGFR‐mutated NSCLC with BMs. The efficacy of afatinib in CNS disease has been reported with an ORR of 72.9% and median CNS PFS of 8.2 months.^9^ Likewise, a large‐scale clinical trial has demonstrated a promising response rate and survival benefit with osimertinib in EGFR‐mutated NSCLC patients with brain metastases, with an ORR of 91% in the cEFR set and 66% in the full‐analysis set.^16^ However, the median PFS was not reached. Comparing above and current studies, the iORR of dacomitinib is comparable to the iORR of osimertinib, meaning that the efficacy of dacomitinib in CNS does not appear to be inferior to that of osimertinib. Nevertheless, the median iDoR and iPFS of our study were not reached, with a 1‐year iDoR rate of 72.2% and a 1‐year iPFS rate of 71.2%. Only two of seven patients with disease progression had intracranial progression, indicating that these responses were durable. As the mature iPFS was lacking, a long‐term follow‐up is needed to verify the survival benefit with dacomitinib in EGFR‐mutated NSCLC patients with brain disease.

The optimal dose of dacomitinib was 45 mg OD, recommended by the dose‐escalation trial.^17^ All patients started taking dacomitinib at 45 mg OD in ARCHER 1050. However, grade 3–4 toxicities were observed frequently compared with gefitinib,^18^ resulting in two‐thirds of patients having dose reduction, with half of them reducing the dose twice.^19^ For this reason, the majority (87.5%) of patients included in this study started on 30 mg OD dacomitinib. Compared to ARCHER 1050, our study had a lower proportion of dose reductions and grade 3‐4 adverse events. However, the difference between patients on 30 mg OD and patients on 45 mg OD in this study was not analyzed, as the population was not balanced between the two groups. Thus, evidence is lacking on this question of the best dose for EGFR‐mutated NSCLC. Deciding on the optimal dose will need to take into account several clinical factors such as performance status, age, and type of EGFR mutation. A clinical trial (NCT04027647), carry out by National Cancer Centre, Singapore, is ongoing to verify the efficacy of dacomitinib with 30 mg OD as first‐line treatment in EGFR‐mutated NSCLC patients.

The optimal management of BMs remains obscure. The BRAIN trial^20^ compared outcomes in patients who received icotinib treatment alone with those who received chemotherapy concurrent with whole brain‐irradiation (WBI) and showed that icotinib was associated with significantly longer intracranial PFS than WBI plus chemotherapy. Considering the limitation of cerebrospinal fluid penetration, first‐generation EGFR TKIs concurrent with brain radiotherapy have attracted much attention. It was reported that patients who received EGFR TKI combined with brain radiotherapy have prolonged survival compared with those who received EGFR TKI alone in a meta‐analysis.^21^ However, data about dacomitinib‐based combination therapy are scarce. A further clinical trial will be needed to explore the best strategy for brain disease in patients with EGFR‐positive NSCLC.

These data are a part of a clinical trial, which is a multicenter observational study with both retrospective and prospective data collection to describe the effectiveness and safety of sequential dacomitinib and third‐generation EGFR TKIs used in patients with EGFR‐mutation‐positive NSCLC with T790M‐acquired resistance in a clinical practice setting. More data about the CNS response to dacomitinib in patients with EGFR‐mutated NSCLC will be reported in the future. There were several limitations in our study. First, this is a single‐arm study that did not compare the efficacy with first‐generation EGFR TKIs in brain metastases. Second, due to the low percentage of patients with measurable brain lesions, there is a lack of sufficient samples to obtain definitive results. Finally, owing to the short follow‐up time, the survival outcome is immature. In the next stage, we will augment the sample size to confirm this result. Further prospective studies will also be needed, such as head‐to‐head clinical trials to find the best strategy for brain disease.

## CONCLUSION

In brief, dacomitinib showed encouraging intracranial activity in patients with EGFR‐positive NSCLC and BMs, with tolerable AEs. Thus, dacomitinib may serve as another treatment option for untreated BMs.

## CONFLICT OF INTEREST

This trial receives funding from Pfizer. However, Pfizer has not been involved in study design, data collection, management, data analysis, and interpretation, or in the decision to submit this article for publication. The authors have stated that they have no conflicts of interest.

## Supporting information


**Table S1**Gene profile and efficacy of dacomitinib in patients with EGFR‐mutated NSCLC and brain metastases
**Table S2** Site of disease progression and EGFR TKI modes in patients who had progressed following dacomitinib treatment
**Table S3** Concordance between central nervous system objective response rate and systemic RECIST objective response rate
**Table S4** Follow‐up treatment in patients who had progressed following dacomitinib treatment
