# Supplementary material for: Efficacy of dacomitinib in patients with EGFR‐mutated NSCLC and brain metastases
Source: Thorac Cancer. 2021 Nov 9;12(24):3407–15. doi: 10.1111/1759-7714.14222 (PMC8671892; doi:10.1111/1759-7714.14222)
Supplement: Supplementary file 1 — Table S1Gene profile and efficacy of dacomitinib in patients with EGFR‐mutated NSCLC and brain metastases Table S2 Site of disease progression and EGFR TKI modes in patients who had progressed following dacomitinib treatment Table S3 Concordance between central nervous system objective response rate and systemic RECIST objective response rate Table S4 Follow‐up treatment in patients who had progressed following dacomitinib treatment [file TCA-12-3407-s001.docx]

**Supplemental table 1：**Gene profile and efficacy of dacomitinib in patients with EGFR-mutated NSCLC and brain metastases

| Patient ID | Gene test method | Specimen for gene test | EGFR mutation | Frequency | Co-mutation | Brain target lesion | CNS response | Systematic response |
| --- | --- | --- | --- | --- | --- | --- | --- | --- |
| P1 | NGS | tissue | L858R | 29.27% | TP53, ALK | Yes | NE* | NE* |
| P2 | NGS | tissue | 19DEL | 7.20% | TP53, NTRK1, CTNNB1 | No | CR | PR |
| P3 | ARMS | tissue | L858R | - | - | No | Non-CR/non-PD | SD |
| P4 | ARMS | tissue | 19DEL | - | - | No | CR | PR |
| P5 | NGS | tissue | L858R | 10.94% | NONE | No | CR | PR |
| P6 | ARMS | tissue | L858R | - | - | Yes | PR | SD |
| P7 | NGS | tissue | L858R | 34.65% | TP53, HER amp, ALK, TSC2, PDGFRA | No | Non-CR/non-PD | PR |
| P8 | NGS | tissue | L858R | 24.01% | PIK3CA | No | CR | PR |
| P9 | NGS | tissue | L858R | 37.41% | TP53, MYC amp | No | CR | PR |
| P10 | ARMS | tissue | L858R | - | - | No | CR | PR |
| P11 | NGS | plasma | L858R | 0.48% | - | No | CR | PR |
| P12 | ARMS | tissue | L858R | - | - | No | CR | Non-CR/non-PD |
| P13 | ARMS | tissue | L858R | - | - | No | Non-CR/non-PD | Non-CR/non-PD |
| P14 | NGS | tissue | L858R | 17.50% | EGFR amp, CDK4 amp, ROS1, CTNNB1, APC, mTOR | Yes | CR | PR |
| P15 | NGS | tissue | L858R, V834L (21 exon) | 50.64%, 51.46% | TP53 nonsense mutation, MYC amp, STK11 amp, PIK3CA amp, KRAS, other mutations | No | Non-CR/non-PD | PR |
| P16 | NGS | tissue | L861Q | 9.30% | TP53, CCND1, BRAF V600E, AR | No | Non-CR/non-PD | Non-CR/non-PD |
| P17 | NGS | tissue | L858R | 11.30% | TP53 | Yes | SD | PR |
| P18 | NGS | pleural effusion | L858R | 25.40% | TP53, FGFR1 | No | CR | SD |
| P19 | NGS | tissue | 19DEL | 46.30% | TP53 | Yes | PR | PR |
| P20 | ARMS | tissue | L858R | - | - | No | CR | PR |
| P21 | ARMS | tissue | L858R | - | - | No | CR | PR |
| P22 | ARMS | tissue | 19DEL | - | - | No | CR | SD |
| P23 | NGS | plasma | L858R | 0.10% | DNMT3A | Yes | NE^+^ | PD |
| P24 | ARMS | tissue | 19DEL | - | - | Yes | PR | PR |
| P25 | ARMS | tissue | L858R | - | - | No | CR | Non-CR/non-PD |
| P26 | NGS | tissue | L858R | 41.80% | TP53, PIK3CA, NF1, IRF2, TERT, APC | Yes | PR | PR |
| P27 | NGS | tissue | L858R | 26.90% | TP53, MYC amp, MDM2, DDR2, NF1, PIK3CA, other mutations | No | Non-CR/non-PD | PR |
| P28 | ARMS | tissue | L858R | - | - | Yes | PR | PR |
| P29 | NGS | tissue | L858R | 75.40% | TP53, EGFR amp, ALK fusion, other mutations | No | Non-CR/non-PD | PR |
| P30 | NGS | plasma | G719A, I759M（19 exon） | 8.4%、9.43% | TP53, EGFR amp, KEAP1, NTRK1 amp, FGFR3, KIT, CDH18 | Yes | PR | PR |
| P31 | NGS | pleural effusion | L858R | 15.10% | TP53, NTRK1 fusion, ALK fusion, DDR2, NF1, JAK1, IGF1R | No | Non-CR/non-PD | SD |
| P32 | ARMS | tissue | L858R | - | - | No | Non-CR/non-PD | PR |

NGS next-generation sequence

*the patient was not evaluable for response owing to lack of baseline examination

^+^the patient was not evaluable for CNS response due to the short interval between brain radiotherapy and starting dacomitinib treatment

**Supplemental table 2:** Site of disease progression and EGFR TKI modes in patients who had progressed following dacomitinib treatment

| **Classification (N=32)** | **N (%)** |
| --- | --- |
| Patients who had progressed following dacomitinib treatment | 7 (21.9) |
| Site of disease progression |  |
| Intracranial | 1 (3.1) |
| Extracranial | 5 (15.6) |
| Extracranial + intracranial | 1 (3.1) |
| EGFR TKI failure mode**^*^** |  |
| Dramatical progression | 3 (9.4) |
| Gradual progression | 2 (6.3) |
| Local progression | 2 (6.3) |

TKI tyrosine kinase inhibitor

**^*^**the assessment criteria for EGFR TKI failure modes included time to disease control, site of disease progression, tumor burden score and clinical symptoms.

**Supplemental table 3:** Concordance between central nervous system objective response rate and systemic RECIST objective response rate

|  | **Systemic ORR** | | |
| --- | --- | --- | --- |
| **CNS ORR** | **Overall responder** | **Overall non-responder** | **Total** |
| All patients (n=30) | (n=21) | (n=9) | (n=30) |
| CNS responder (n=20), n (%) | 15 (50.0) | 5 (16.7) | 20 (66.7) |
| CNS non-responder (n=10), n (%) | 6 (20.0) | 4 (13.3) | 10 (33.3) |
| Total (n=30), n (%) | 21 (70.0) | 9 (30.0) | 30 (100) |

**Supplemental table 4:** Follow-up treatment in patients who had progressed following dacomitinib treatment

| **Classification (N=32)** | **N (%)** |
| --- | --- |
| Patients who received subsequent anti-cancer therapy after progression | 6 (18.75) |
| The third generation EGFR-TKI | 2 (6.3) |
| T790M negative | 0 (0) |
| T790M positive | 2 (6.3) |
| Dacomitinib combined with bevacizumab | 1 (3.1) |
| Platinum-based chemotherapy combined with bevacizumab | 2 (6.3) |
| Dose intensification of dacomitinib (45mg OD) | 1 (3.1) |
